# High-mobility group box-1 impedes skeletal muscle regeneration via downregulation of Pax-7 synthesis by increasing miR-342-5p expression

**DOI:** 10.18632/aging.205202

**Published:** 2023-11-13

**Authors:** Trung-Loc Ho, Yu-Liang Lai, Chin-Jung Hsu, Chen-Ming Su, Chih-Hsin Tang

**Affiliations:** 1 Graduate Institute of Biomedical Sciences, College of Medicine, China Medical University, Taichung, Taiwan; 2 Department of Physical Medicine and Rehabilitation, China Medical University Hsinchu Hospital, Hsinchu, Taiwan; 3 Department of Physical Therapy and Graduate Institute of Rehabilitation Science, China Medical University, Taichung, Taiwan; 4 Department of Physical Medicine and Rehabilitation, China Medical University Hospital, Taichung, Taiwan; 5 School of Chinese Medicine, China Medical University, Taichung, Taiwan; 6 Department of Orthopedic Surgery, China Medical University Hospital, Taichung, Taiwan; 7 Department of Sports Medicine, China Medical University, Taichung, Taiwan; 8 Department of Pharmacology, School of Medicine, China Medical University, Taichung, Taiwan; 9 Chinese Medicine Research Center, China Medical University, Taichung, Taiwan; 10 Department of Medical Laboratory Science and Biotechnology, College of Health Science, Asia University, Taichung, Taiwan; 11 Department of Medical Research, China Medical University Hsinchu Hospital, Hsinchu, Taiwan

**Keywords:** muscle regeneration, HMGB1, Pax-7, myoblasts, miR-342-5p

## Abstract

High mobility group box-1 (HMGB1) is a driver of inflammation in various muscular diseases. In a previous study, we determined that HMGB1 induced the atrophy of skeletal muscle by impairing myogenesis. Skeletal muscle regeneration after injury is dependent on pair box 7 (Pax-7)-mediated myogenic differentiation. In the current study, we determined that the HMGB1-induced downregulation of Pax-7 expression in myoblasts inhibited the regeneration of skeletal muscle. We also determined that HMGB1 inhibits Pax-7 and muscle differentiation by increasing miR-342-5p synthesis via receptors for advanced glycation end-products (RAGE), toll-like receptor (TLR) 2, TLR4, and c-Src signaling pathways. In a mouse model involving glycerol-induced muscle injury, the therapeutic inhibition of HMGB1 was shown to rescue Pax-7 expression and muscle regeneration. The HMGB1/Pax-7 axis is a promising therapeutic target to promote muscular regeneration.

## INTRODUCTION

Muscle regeneration is a complex process involving the formation of new muscle fibers after injury [1–3]. Muscle regeneration relies on myogenesis, which involves the activation, proliferation, and differentiation of satellite cells or myoblasts [4]. The fusing of myoblasts into multinucleated myotubes results in the enlargement of myofibers, as indicated by the expression of differentiation markers, such as myosin heavy chain (MyHC) [5], desmin [6], or myogenin (MyoG) [7]. Activating myoblast proliferation and regeneration requires various myogenic markers, including myogenic regulatory factors such as pair box 7 (Pax-7), myogenic regulatory factor 4 (Mrf4), and myogenic factor 5 (Myf5) [8, 9]. Pax-7 has been shown to regulate the conversion of muscle stem cells into myoblasts and participate in other processes related to myogenesis [10]. Pax-7 activation plays a key role in the formation of new muscle fibers after injury [11]. These findings indicate that Pax-7 dysfunction could inhibit the differentiation of skeletal muscle and contribute to impairing muscle regeneration.

MicroRNAs (miRNAs) are a class of small non-coding RNAs that play an essential role in regulating skeletal muscle homeostasis and responding to muscle injury [12, 13]. Myoblast proliferation and differentiation depend on a variety of miRNAs, including miR-22 [14], miR-19 [15], miR-29c [16], and miR-324-5p [13]. However, the role of miRNAs in regulating Pax-7-induced muscle regeneration remains unclear.

High mobility group box-1 (HMGB1) is a ubiquitous nuclear protein that potentiates cellular inflammation [17]. Compared to normal individuals, HMGB1 levels in skeletal muscle are markedly higher in the serum of patients with muscular dystrophy [18] or inflammatory myopathy [19]. Researchers have detected the upregulation of the HMGB1 gene in a mouse model involving denervation-induced muscle atrophy [20]. Researchers have also demonstrated that in patients with myositis, the interaction of HMGB1 with receptors for advanced glycation end-products (RAGE) or toll-like receptor (TLR) 2 and TLR4 induced the wasting of muscle tissue [21–24]. In a previous study, we demonstrated that HMGB1 treatment impeded myogenesis in myoblast cells. We also observed elevated HMGB1 protein expression in a mouse model involving glycerol-induced muscle injury (GIMI). The oxidation of HMGB1 has been implicated in the development of the dystrophic phenotype, resulting in inflammation and muscle degeneration [18]. HMGB1 can be used as an indicator of muscle atrophy; however, researchers have yet to determine whether the inhibited regeneration of skeletal muscle is due to the HMGB1-induced downregulation of Pax-7 expression in myoblasts.

## RESULTS

### HMGB1 inhibits Pax-7 expression and muscle differentiation

**Figure 1:**
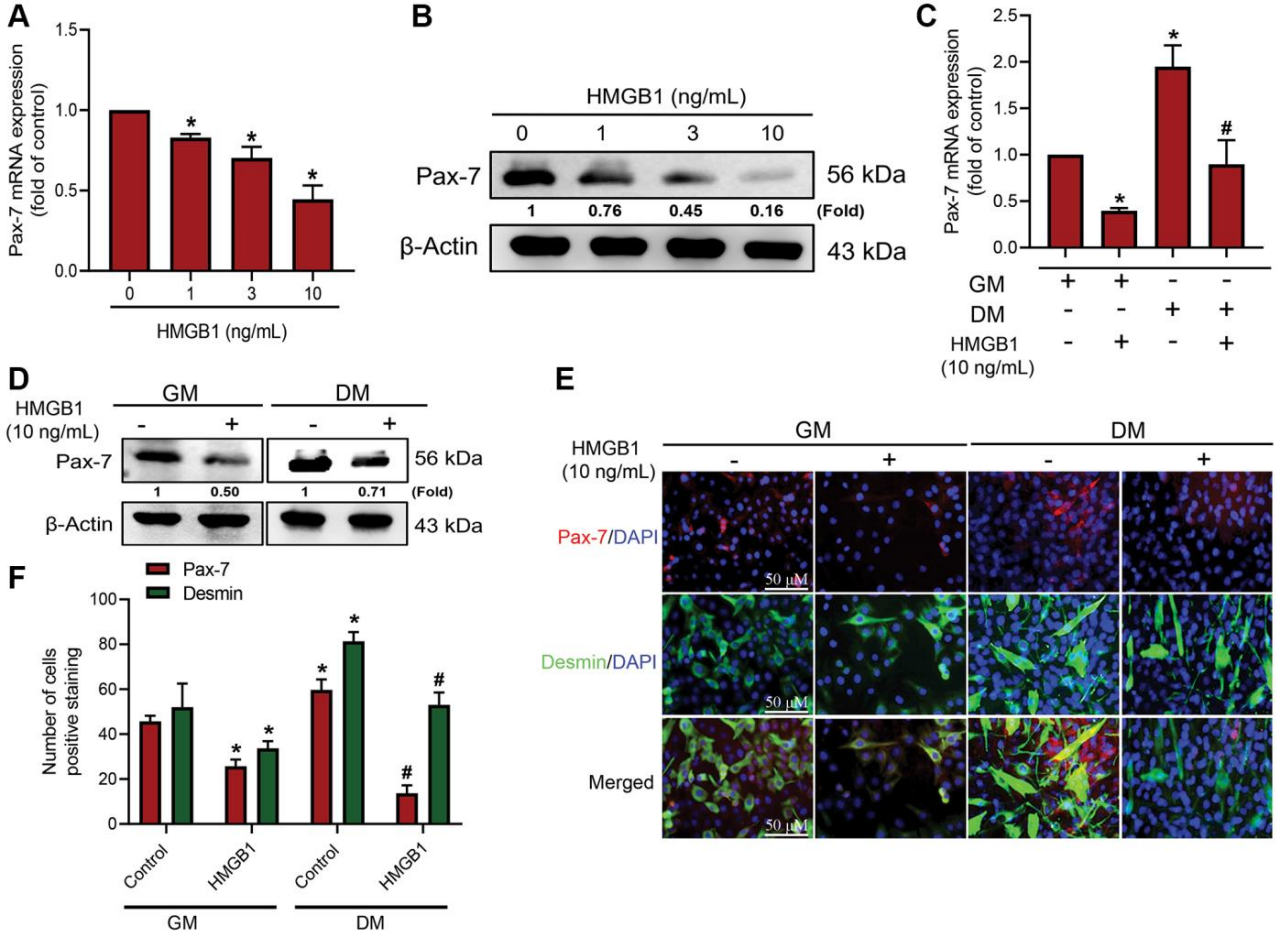
**HMGB1 inhibits Pax-7 synthesis in skeletal muscle myoblasts.** After exposing C2C12 cells to HMGB1 for 24 h, the expression of Pax-7 mRNA and protein was examined by (**A**) qRT-PCR (*n* = 3) and (**B**) western blotting assay (*n* = 3). C2C12 cells were cultured in growth medium (GM) and differentiation medium (DM) for three days with or without HMGB1 (10 ng/mL) for 24 h, after which Pax-7 expression was examined using (**C**) qRT-PCR (*n* = 3) and (**D**) western blotting assay (*n* = 3). (**E**) Immunofluorescence staining showing the expression of Pax-7 and desmin in C2C12 cells cultured in GM and DM after HMGB1 treatment (n = 3). Pax-7 is indicated by red coloring, desmin is indicated by green, and DAPI is shown in blue. Scale bars = 50 μm. (**F**) Number of positively stained cells were quantified by ImageJ software (*n* = 3). β-Actin was used as the loading control. All data are presented as the mean ± SD of triplicate experiments. ^*^p < 0.05 compared with control group; ^#^*p* < 0.05 compared with HMGB1-treated group. Abbreviations: GM: growth medium; DM: differentiation medium.

Considering the importance of Pax-7 as a marker in muscle regeneration, our objective in the current study was to determine whether HMGB1 affects Pax-7 expression in myoblasts. This was achieved by treating myoblasts with various concentrations of mouse recombinant HMGB1 protein over a period of 24 h. qRT-PCR analysis and western blot assays revealed that HMGB1 significantly inhibited Pax-7 mRNA expression and protein expression, respectively (Figure 1A, 1B). We also explored the effects of HMGB1 on myoblast differentiation through the use of differentiation medium (DM) to initiate the development to myotubes. After three days of DM induction, it was observed that HMGB1 suppressed Pax-7 mRNA and Pax-7 protein expression (Figure 1C, 1D). We also examined the role of Pax-7 in HMGB1-induced muscle regeneration using immunofluorescence double-staining to detect the expression of differentiation markers (Pax-7 and desmin) in GM and DM (Figure 1E). Our results revealed that HMGB1 inhibited differentiation (Pax-7 and desmin expression) in GM and DM (Figure 1F), thereby indicating that HMGB1 suppressed the synthesis of Pax-7, which in turn impaired muscle differentiation activity.

### RAGE, TLR2, and TLR4 are involved in HMGB1-inhibited Pax-7 expression and muscle differentiation

**Figure 2:**
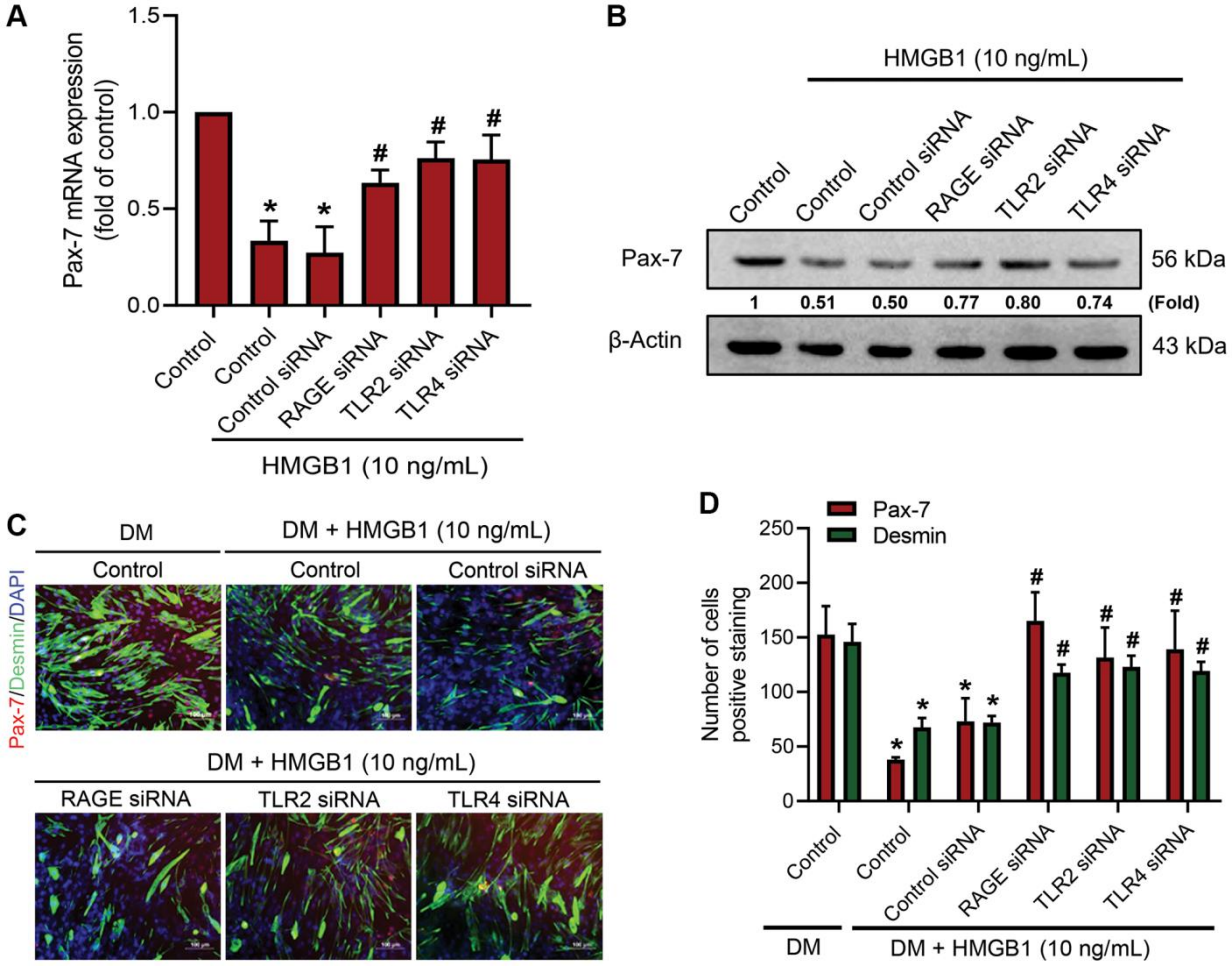
**RAGE, TLR2, and TLR4 receptors are involved in the downregulation of Pax-7 expression by HMGB1.** C2C12 cells were pre-treated with 100 nM siRNA against RAGE, TLR2, or TLR4 for 24 h and then subjected to HMGB1 (10 ng/mL) stimulation for 24 h. The expression levels of Pax-7 mRNA and protein were respectively analyzed by (**A**) qRT-PCR (*n* = 3) and (**B**) western blotting assay (*n* = 3). (**C**) Immunofluorescence staining was used to visualize the expression of Pax-7 and desmin in C2C12 cells in DM after HMGB1 treatment (10 ng/mL) with indicated siRNAs (*n* = 3). Pax-7 is indicated by red coloring, desmin is indicated by green, and DAPI is shown in blue. Scale bars = 100 μm. (**D**) Number of positively stained cells were quantified by ImageJ software (*n* = 3). β-Actin was used as the loading control. All data are presented as the mean ± SD of triplicate experiments. ^*^p < 0.05 compared with control group; ^#^*p* < 0.05 compared with HMGB1-treated group.

Previous studies have shown that RAGE, TLR2, and TLR4 are associated with HMGB1-induced cellular activation [25, 26]. In this study, we sought to identify the receptors that play an important role in the HMGB1-induced inhibition of Pax-7 expression and muscle differentiation. The use of small interfering RNA (siRNA) to knockdown the expression of RAGE, TLR2, and TLR4 receptors was shown to reverse the HMGB1-induced inhibition of Pax-7 mRNA expression (Figure 2A) and protein expression (Figure 2B). Immunofluorescence assays provided further evidence indicating the involvement of RAGE, TLR2, and TLR4 in the HMGB1-induced decrease of muscle differentiation (Figure 2C, 2D). Our results demonstrate that RAGE as well as TLR2 and TLR4 receptors are involved in HMGB1 impairing Pax-7 expression and muscle differentiation.

### c-Src signaling is involved in HMGB1-mediated Pax-7 expression and muscle differentiation

**Figure 3:**
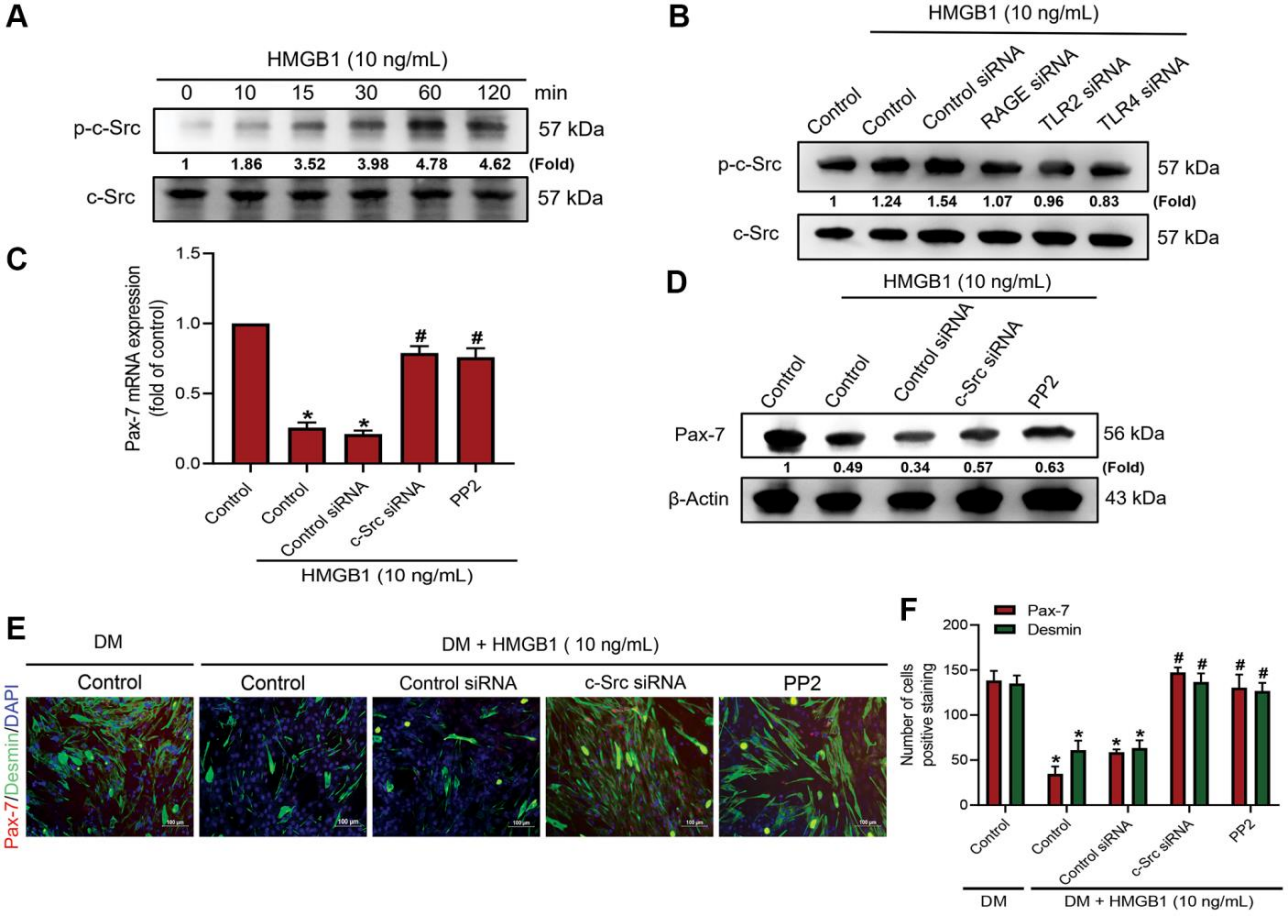
**c-Src signaling is involved in HMGB1-mediated Pax-7 expression and muscle differentiation.** Western blot analysis of c-Src phosphorylation in (**A**) C2C12 cells treated with HMGB1 (10 ng/mL) following a time-dependent manner (*n* = 3) and (**B**) C2C12 cells pre-treated with various siRNAs against RAGE, TLR2, and TLR4 molecules (100 nM) and then exposed to HMGB1 (10 ng/mL) for 24 h. (**C**) qRT-PCR (*n* = 3) and (**D**) western blot analysis of Pax-7 mRNA and protein expression, respectively, in C2C12 cells after HMGB1 co-treatment with c-Src siRNA (100 nM) or c-Src inhibitor (PP2; 1 μM) for 24 h (*n* = 3). (**E**) Immunofluorescence staining to visualize the expression of Pax-7 and desmin in C2C12 cells in DM after HMGB1 (10 ng/mL) co-treatment with c-Src siRNA (100 nM) or PP2 (1 μM) for 24 h (*n* = 3). Pax-7 is indicated by red coloring, desmin is indicated by green, and DAPI is shown in blue. Scale bars = 100 μm. (**F**) Number of positively stained cells were quantified by ImageJ software (*n* = 3). β-Actin was used as the loading control. All data are presented as the mean ± SD of triplicate experiments. ^*^p < 0.05 compared with control group; ^#^*p* < 0.05 compared with HMGB1-treated group.

One previous study reported that the suppression of cellular Src kinase (c-Src) activity is involved in regulating muscle differentiation [27]. Our objective in the current study was to determine whether c-Src is involved in the HMGB1-induced inhibition of Pax-7 synthesis and skeletal muscle differentiation. We found that incubating myoblasts with 10 ng/mL of HMGB1 significantly enhanced the phosphorylation of c-Src (Figure 3A), the effects of which were abolished by RAGE, TLR2, or TLR4 siRNA (Figure 3B). Pre-treating cells with a c-Src siRNA or c-Src inhibitor (PP2) was shown to restrict the HMGB1-induced decrease in Pax-7 expression (Figure 3C, 3D). Inhibiting c-Src signaling was also shown to reverse the effects of HMGB1 on muscle differentiation (Figure 3E, 3F). These findings suggest that the effects of HMGB1 on Pax-7 expression and muscle differentiation involve the activation of c-Src signaling via the RAGE, TLR2, and TLR4 receptors.

### HMGB1 suppresses Pax-7 synthesis and muscle differentiation by promoting miR-342-5p expression

**Figure 4:**
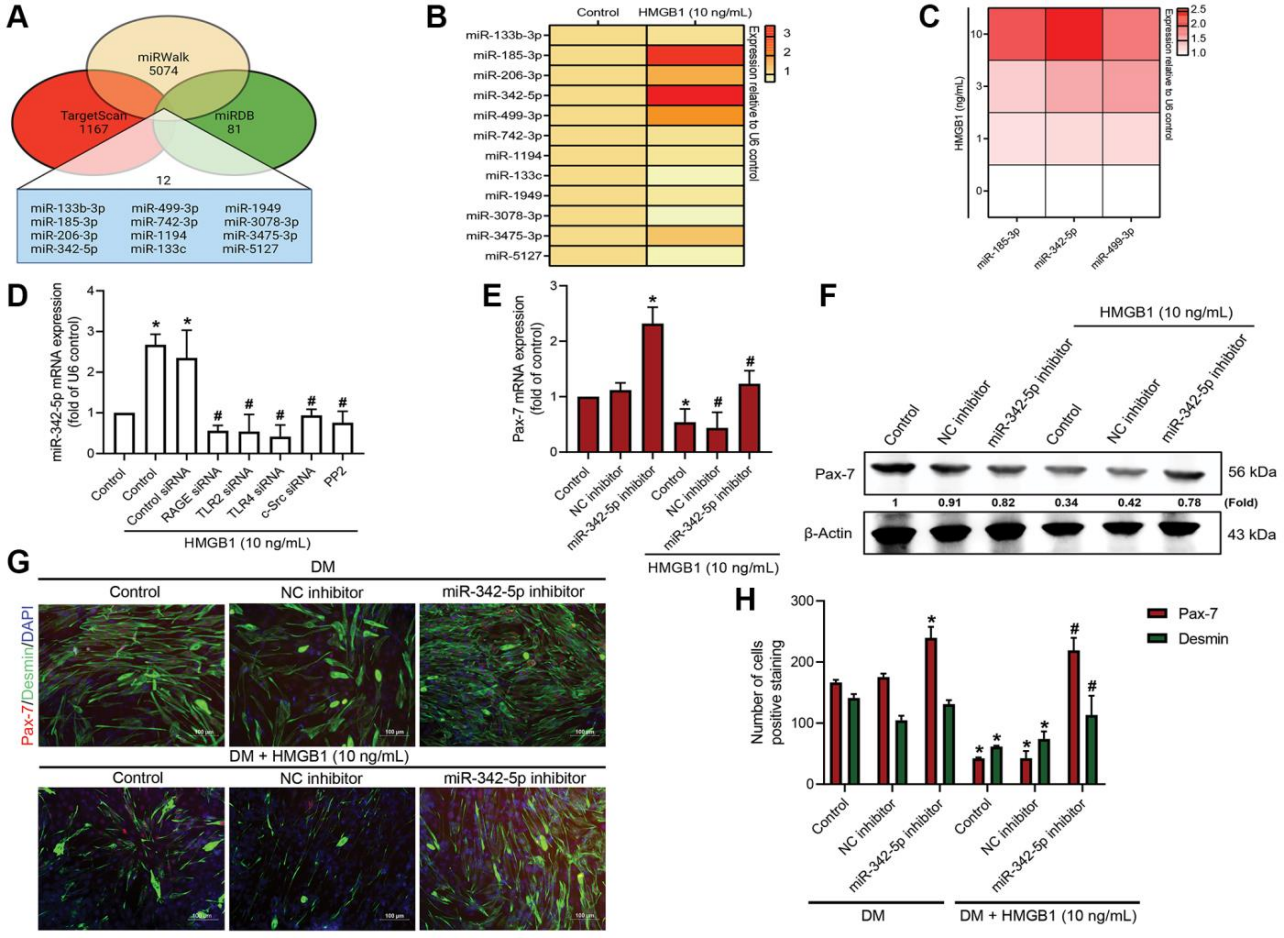
**miR-342-5p/Pax-7 axis is involved in HMGB1-induced inhibition of skeletal muscle differentiation.** (**A**) Potential miRNA targets of Pax-7 predicted by miRWalk, miRDB, and TargetScan. (**B**) qRT-PCR assays showing the expression of the 12 predicted miRNAs following HMGB1 treatment (10 ng/mL) (*n* = 3). (**C**) qRT-PCR assays showing the expression of miR-185-3p, miR-342-5p, and miR-499-3p in C2C12 cells after HMGB1 treatment (*n* = 6). (**D**) qRT-PCR assays showing miR-342-5p expression in C2C12 cells transfected with various siRNAs or pre-treated with c-Src inhibitor (PP2; 1 μM) prior to HMGB1 treatment for 24 h (*n* = 3). (**E**) qRT-PCR analysis and (**F**) western blot analysis showing the effect of miR-342-5p inhibitor on Pax-7 mRNA expression and protein expression (*n* = 3), respectively. (**G**) Immunofluorescence staining showing the expression of Pax-7 and desmin in C2C12 cells in DM after HMGB1 treatment (10 ng/mL) with 50 nM of miR-342-5p inhibitor or negative control (*n* = 3). Pax-7 is indicated by red coloring, desmin is indicated by green, and DAPI is shown in blue. Scale bars = 100 μm. (**H**) Number of positively stained cells were quantified by ImageJ software (*n* = 3). β-Actin was used as the loading control. All data are presented as the mean ± SD of triplicate experiments. ^*^p < 0.05 compared with control group; ^#^*p* < 0.05 compared with HMGB1-treated group.

Research in the field of bioinformatics has revealed that over 30% of the proteins in mammals are regulated by miRNAs [28]. In the current study, we employed publicly available bioinformatics software (TargetScan, miRWalk, and miRDB) to identify the miRNAs that are involved in Pax-7 expression. Cross-checking the three programs revealed 12 miRNAs that bind directly to Pax-7 (Figure 4A). A correlation heat map revealed that miR-185-3p, miR-342-5p, and miR-499-3p were highly expressed in HMGB1-treated myoblasts, leading to their selection for further study (Figure 4B). Our analysis revealed that HMGB1 enhanced the expression of miR-342-5p in a dose-dependent manner (Figure 4C). The HMGB1-induced promotion of miR-342-5p expression was moderated by the presence of RAGE, TLR2, TLR4, c-Src, indicating that miR-342-5p acted downstream from these molecules (Figure 4D). We also sought to determine whether the HMGB1-induced effects on Pax-7 expression depended on miR-342-5p synthesis. Transfecting a miR-342-5p inhibitor into myoblasts was shown to reverse the HMGB1-induced reduction in Pax-7 expression and muscle differentiation (Figure 4E–4H). Taken together, it appears that HMGB1 reduces Pax-7 expression and muscle differentiation by promoting the synthesis of miR-342-5p via RAGE, TLR2, TLR4, and c-Src signaling cascades.

### Inhibition of HMGB1 reverses GIMI and Pax-7 expression

**Figure 5:**
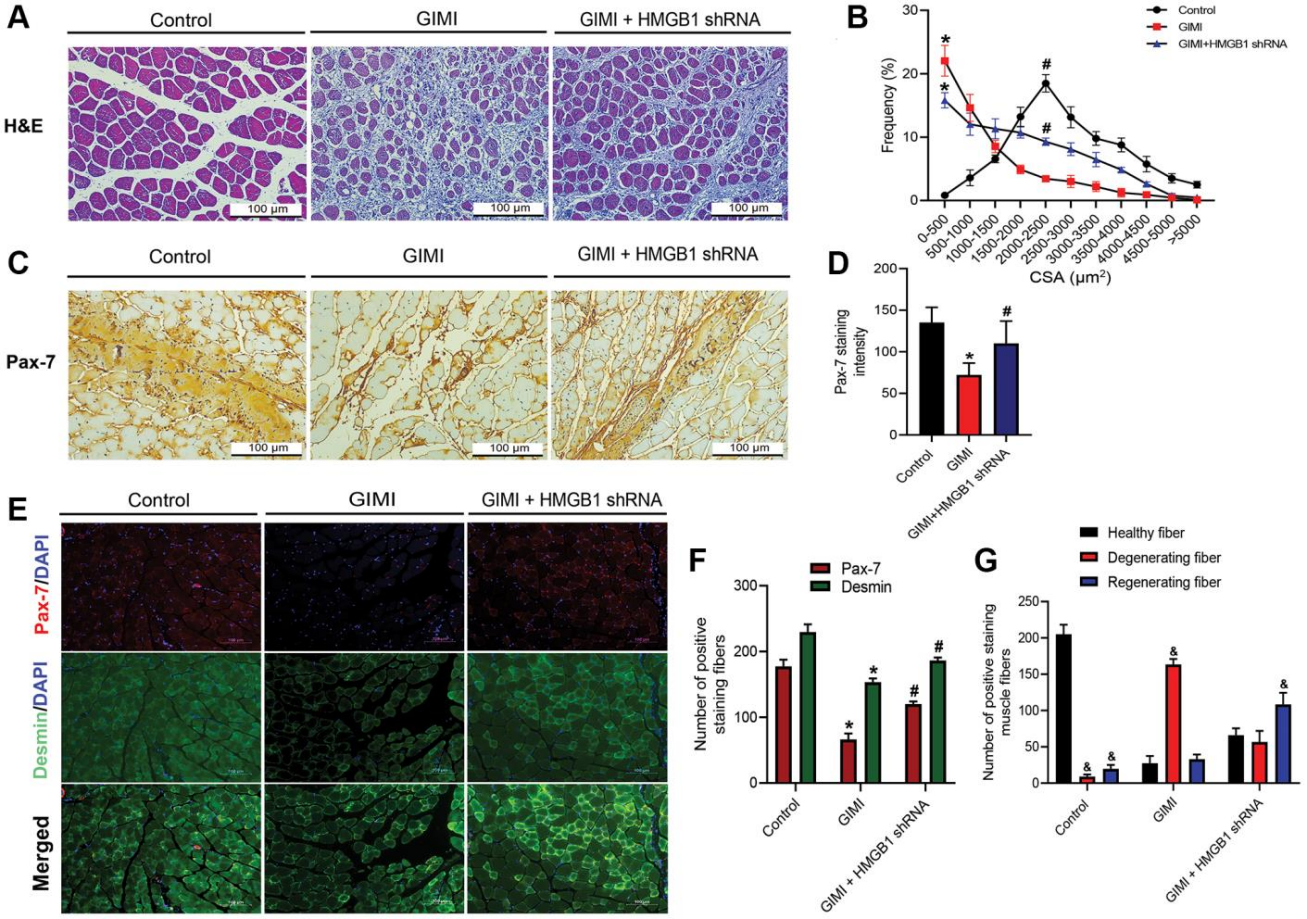
**Inhibition of HMGB1 reverses glycerol-induced muscle injury (GIMI) and Pax-7 expression.** (**A**) Representative images of H&E-stained tibialis anterior (TA) sections from mice in control group and glycerol-induced muscle injury (GIMI) without or with HMGB1 shRNA treated-group (GIMI+HMGB1 shRNA) (*n* = 3); scale bars = 100 μm. (**B**) Frequency distribution of cross-sectional area (CSA) of TA muscle fibers (*n* = 3). (**C**) Immunohistochemistry analysis of Pax-7 protein expression in TA muscle of control group, GIMI group, and GIMI+HMGB1 shRNA group (*n* = 3). (**D**) Quantification of Pax-7 staining intensity in TA muscle (*n* = 6). (**E**) Immunofluorescence analysis showing the expression of Pax-7 and desmin in TA muscle in the control, GIMI, and GIMI+HMGB1 shRNA groups (n = 3); scale bars = 100 μm. (**F**) Quantification of the Pax-7 and desmin positive staining fibers in TA muscle were analyzed by ImageJ software (*n* = 3). (**G**) The healthy fiber (large cells), degenerating fiber (mononuclear in necrotic cells), and regenerating fiber (multinucleated cells) were quantified with the positive double-staining of Pax-7 and desmin by using ImageJ software (*n* = 3). All data are presented as the mean ± SD of triplicate experiments. ^&^*p* < 0.05 compared with the healthy fiber; ^*^p < 0.05 compared with control group; ^#^*p* < 0.05 compared with GIMI group.

Intramuscular glycerol injection is a recent approach to inducing muscle injury and subsequent regeneration [29–31]. We previously reported that GIMI can be rescued by inhibiting the effects of HMGB1 using HMGB1 shRNA [32]. H&E staining was used to show the cross-sectional area (CSA) of muscle fibers from the tibialis anterior (TA) (Figure 5A). We then measured the distribution of CSA in the control group, GIMI group, and GIMI+HMGB1 shRNA group. These results indicate that the increase in CSA was more pronounced in the GIMI+HMGB1 shRNA group than in the GIMI group (Figure 5B). HMGB1 shRNA was also shown to restore the protein expression of Pax-7 in skeletal muscle to normal levels (Figure 5C, 5D). The role of Pax-7 in myogenesis was assessed *in vivo* by examining muscle regeneration via immunocytochemical staining. We found that a decrease in Pax-7 protein expression was correlated with desmin in TA muscle fibers following glycerol injury. Both proteins presented elevated expression levels in the HMGB1 shRNA-treated group (Figure 5E, 5F). In addition, the increasing number of regenerating fiber correlates to the expression of Pax-7 and desmin proteins (Figure 5G). These results indicate that inhibiting HMGB1 ameliorated glycerol-induced muscle damage and subsequent muscle regeneration by restoring Pax-7 expression.

## DISCUSSION

It has been established that the regenerative capacity of skeletal muscle involves differentiation and myogenesis [33–36]. Recent research has revealed links between aberrant HMGB1 expression and muscle wasting or dysfunction [19, 37, 38]. In prior investigations, we substantiated that HMGB1, at a concentration of 10 ng/mL, has the capacity to exacerbate skeletal muscle atrophy by impeding various facets of myogenic processes [32]. It has been well-documented that necrotic and immune cells prominently contribute to the active or passive release of HMGB1 into the extracellular milieu [17, 39]. This phenomenon is closely associated with the heightened expression of HMGB1 levels during conditions of muscle loss [40]. Nonetheless, the role of HMGB1 in the regeneration of skeletal muscle has yet to be elucidated. In the current study, we found that HMGB1 inhibits the regeneration of skeletal muscle by downregulating Pax-7 expression and myoblast differentiation capacity. Our qRT-PCR results revealed that HMGB1 treatment increased miR-342-5p expression in skeletal muscle cells. We also sought to determine whether the link between inhibited Pax-7 expression and decreased muscle differentiation was associated with HMGB1 receptor signaling (RAGE, TLR2, TLR4) or c-Src signaling. This was achieved via treatment with siRNA against RAGE, TLR2, TLR4, c-Src and a c-Src inhibitor. Our results revealed that miR-342-5p acted as a downstream signaling molecule of RAGE, TLR2, TLR4, and c-Src signaling cascades. HMGB1 blockade was shown to promote the regeneration of skeletal muscle in a mouse model involving induced muscular injury. This study provides solid evidence indicating that the inhibition of HMGB1 signaling is a promising therapeutic approach to promoting muscle regeneration. In consistent with our findings, another research group has also shown that the inhibition of HMGB1 oxidative activity expedites the process of regeneration without aggravating inflammation in both muscle and liver tissues, which serve as pivotal paradigms in the fields of regenerative medicine and biology [18, 41]. Interestingly, therapeutic strategies against these specific HMGB1 isoforms can serve as models for more efficient therapeutic strategies against tissue damage [42, 43]. Taken together, the evidence suggests that HMGB1 represents a novel signaling molecule deserving of targeted intervention for the enhancement of muscle regeneration.

Previous studies have reported that Pax-7 is a switch molecule capable of inducing the regeneration of skeletal muscle following acute muscle injury [44, 45]. The fact that Pax-7 knockout mice presented a diminished number of muscle satellite cells and impaired muscle regeneration supports the notion that Pax-7 is required for the propagation and function of the satellite cell population [46, 47]. We can therefore surmise that Pax-7 plays an essential role in the activation of skeletal satellite cells; however, the role of Pax-7 expression in myoblast differentiation remains unknown. In the current study, *in vitro* analysis revealed that the expression of Pax-7 markers upregulated the differentiation of skeletal muscle, whereas *in vivo* analysis revealed that Pax-7 expression levels were significantly lower in the muscular atrophy group than in the control group. It was also observed that positive staining for Pax-7 in satellite muscle cells was higher in the HMGB1 shRNA treated-group than in the GIMI group (Figure 5C, 5D). From this, we can deduce that the therapeutic knock-down of HMGB1 signaling ameliorated muscular injury by promoting the expression of Pax-7 regeneration markers.

Notably, a previous histological finding indicated that the degenerative changes resulting from glycerol-induced injury bear a striking resemblance to the pathological features observed in Duchenne muscular dystrophy [48]. The intramuscular administration of glycerol has been empirically shown to induce myofiber injury characterized by a degenerative phenotype, as evidenced in rats within a three-day post-injury period [49]. These compelling observations suggest the potential utility of glycerol as a chemical agent for inducing muscle injuries in murine models. Therefore, we used the utilization of intramuscular injection of glycerol as an innovative experimental model for inducing acute muscle injury and triggering skeletal muscle inflammation. The TA muscle has attracted substantial interest as a prominent choice in skeletal muscle injury models, owing to its easily accessible anatomical location and its capacity to consistently produce uniform injury outcomes within 24 h after glycerol injection [48, 50]. Our results demonstrated that glycerol significantly induced TA muscle injury as well as degeneration phenotype in mouse model (Figure 5A, 5B). These outcomes imply that the TA muscle serves as a favourable locus for the induction of muscle injury.

RAGE, TLR2, and TLR4 are the most important HMGB1 receptors affecting the inflammatory response in many cellular diseases [51, 52]. In muscular diseases, RAGE upregulation has been shown to induce atrophy in skeletal muscle [53]. The pharmacological inhibition of TLR4 has been shown to decrease lipopolysaccharide-induced muscle wasting by suppressing the activation of inflammatory and proteolytic pathways [54]. In a previous mouse model, TLR2 knockout was shown to attenuate cardiotoxin-induced atrophy in skeletal muscle [55]. In the current study, the inhibition of RAGE, TLR2, and TLR4 signaling via HMGB1 treatment restored Pax-7 mRNA and protein expression in myoblasts. Taken together, these results confirmed the participation of RAGE, TLR2, and TLR4 receptor signaling in the HMGB1-induced inhibition of myoblast differentiation and Pax-7 synthesis. These results also suggest that the expression of RAGE, TLR2, or TLR4 negatively regulates the regeneration of skeletal muscle.

It is well-known that miRNAs play an essential role in skeletal muscle development via their participation in myogenesis [56], while aberrant miRNA expression has been observed in muscle diseases, including cardiac and skeletal muscle hypertrophy, heart failure, and muscular dystrophy [57–59]. The expression of muscle-specific miRNAs, such as miR-1 and miR-206, is insufficient to induce differentiation in myoblast cells. Knockdown of endogenous miR-1 and miR-206 in a neonatal mouse model was shown to promote the proliferation of satellite cells and increase Pax-7 protein levels in skeletal muscle [60]. In the current study, the upregulation of miR-342-5p by HMGB1 suppressing Pax-7 expression and skeletal muscle cell differentiation suggests that miR-342-5p plays an important role in regulating muscle regeneration via Pax-7. Given the substantial dose-dependent elevation of miR-342-5p observed in myoblast cells following treatment with HMGB1 (Figure 4B, 4C), it is reasonable to postulate that miR-342-5p represents a primary target of HMGB1 action. Nonetheless, our examination of the role of miR-342-5p was limited to *in vitro* analysis. Further analysis will be needed to investigate the role of miR-342-5p *in vivo* in animal models involving muscular injury.

**Figure 6:**
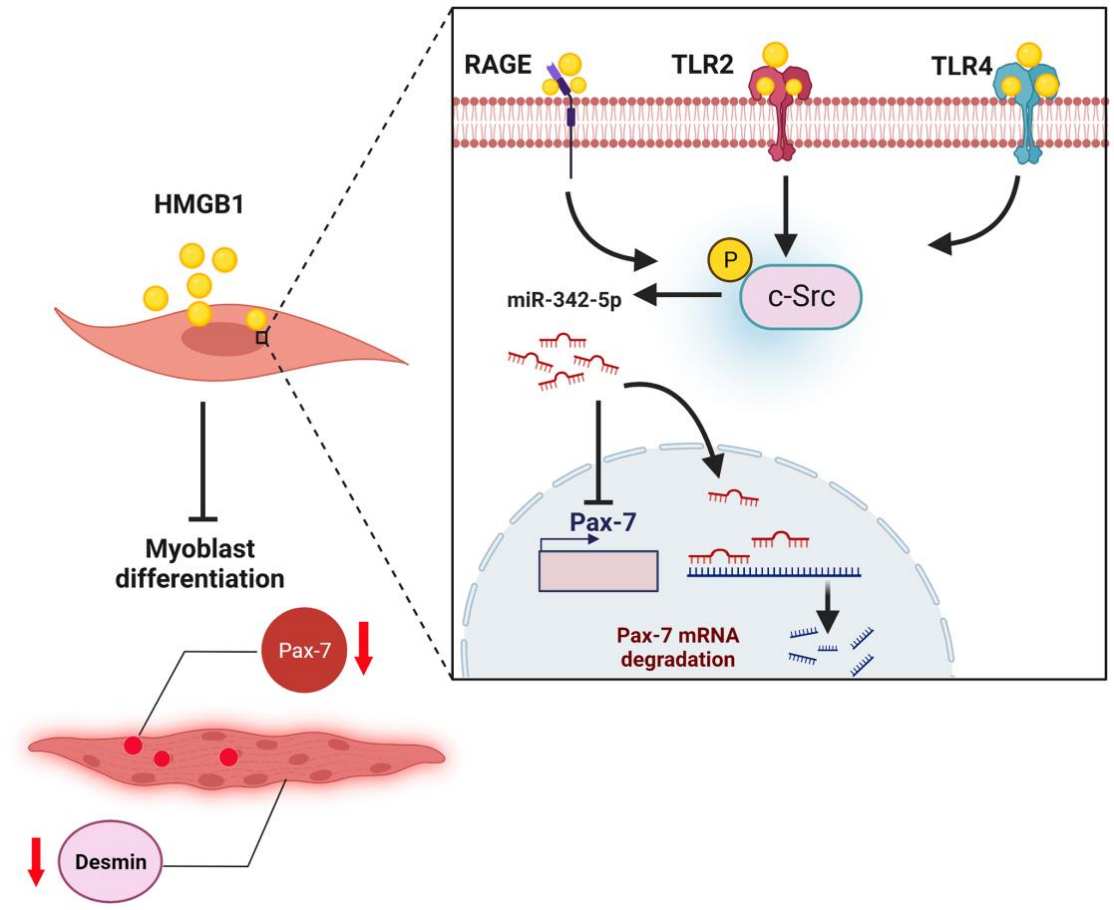
Graphical abstract: schematic illustration of proposed mechanisms underlying the HMGB1-induced inhibition of muscle regeneration, where HMGB1 inhibits Pax-7 expression and skeletal muscle regeneration by promoting the synthesis of miR-342-5p via RAGE, TLR2, TLR4, and c-Src signaling pathways.

In conclusion, this study demonstrated that the HMGB1-induced inhibition of Pax-7 expression impaired skeletal muscle regeneration by promoting the synthesis of miR-342-5p via RAGE, TLR2, TLR4, and c-Src signaling cascades (Figure 6). The therapeutic inhibition of HMGB1 signaling was shown to enhance the regeneration of skeletal muscle by increasing the expression of Pax-7 markers *in vivo*. Our findings suggest that HMGB1 plays key roles in the wasting and regeneration of skeletal muscle.

## MATERIALS AND METHODS

### Reagents and materials

RAGE (sc-36374), TLR2 (sc-40256), TLR4 (sc-40260), c-Src (sc-29228) siRNAs were obtained from Santa Cruz Biotechnology (Dallas, TX, USA); control siRNA (D-001810-10-05) was purchased from Dharmacon (Lafayette, CO, USA). Recombinant mouse HMGB1 protein was obtained from Biorbyt (orb57027, Cambridge, UK). Pax-7 antibodies were obtained from R&D Systems (NBP2-32894, Minneapolis, MN, USA). Antibodies against c-Src (sc-5266) and p-c-Src (sc-12928-R) were obtained from Santa Cruz Biotechnology (Dallas, TX, USA). Desmin antibodies were obtained from Abcam (ab216616, Eugene, OR, USA). Secondary antibodies goat anti-rabbit Alexa Fluor 488 (#A32731) and rabbit anti-mouse Alexa Flour 594 (#A27027) were obtained from Thermo Fisher Scientific (Waltham, MA, USA). PP2 inhibitors (purity > 98%, #CAS 172889-27-9) and DAPI dye (#CAS D9564) were purchased from Sigma-Aldrich (St. Louis, MO, USA). miR-342-5p inhibitor (#65522) and NC miRNA inhibitor (#135060) were purchased from AllBio Science (Taichung City, Taiwan). Mouse shRNA HMGB1 plasmid (Cat#TRCN0000365913) was purchased from National RNAi Core Facility (Sinica, Taiwan), and puro-HMGB1 shRNA-pMD-CMV constructs were purchased from Addgene (Watertown, MA, USA). All *in vivo* reagents and materials were referred to our previous study [32].

### Cell cultures and muscle differentiation

C2C12 myoblasts obtained from the American Type Culture Collection (ATCC, Manassas, VA, USA) [32] were cultured in a growth medium (GM) of Dulbecco’s modified Eagle medium (DMEM) supplemented with 10% fetal bovine serum and penicillin (100 U/mL) (Thermo Fisher Scientific, Waltham, MA, USA). The cells were differentiated into myotubes by changing the growth medium to differentiation medium (DM) for 72 h [61, 62], which consisted of DMEM containing 2% horse serum (Thermo Fisher Scientific, Waltham, MA, USA).

### Transfection assay

C2C12 cells were transfected with RAGE, TLR2, TLR4, c-Src, and control siRNAs or miR-342-5p inhibitors or negative control (NC) miRNA inhibitors by using Lipofectamine2000™ reagent (Thermo Fisher Scientific, Waltham, MA, USA) for 20 min at room temperature according to the manufacturer’s instructions. The mixtures were then applied to the cells in a volume of Opti-MEM™ I (Gibco, Grand Island, NY, USA), giving a final concentration of 100 nM siRNA and 50 nM miRNA-inhibitor for 24 h.

### Quantitative Real-time Polymerase Chain Reaction (qRT-PCR) analysis of mRNA and miRNA

Total RNA was extracted from myoblasts using TRIzol™ reagent (MDBio, Taipei, Taiwan) in accordance with the manufacturer’s instructions. qRT-PCR analysis was performed using the methods outlined in our previous reports [32, 63]. Briefly, 1 μg of total RNA was reverse transcribed to complementary DNA (cDNA) with the oligo-DT primer. cDNA was synthesized using the MMLV reverse transcriptase system (Invitrogen, Carlsbad, CA, USA). miRNA was synthesized using the Mir-X™ miRNA First-Strand Synthesis kit (Terra Bella Avenue, Mountain View, CA, USA) in accordance with the manufacturer’s instructions. qRT-PCR analysis was performed using the Fast SYBR^®^ Green Mix in the StepOnePlusTM system (Applied Biosystems, Foster City, CA, USA). The primers used in this study are listed in Supplementary Table 1. Glyceraldehyde-3-phosphate dehydrogenase (GAPDH) mRNA was used as reference for mRNA quantification, and U6 small nuclear RNA (U6 snRNA) was used for miRNA. The 2^−ΔΔ*CT*^ method was used to quantify the qRT-PCR analyses [64]. The result of this method is presented as the fold change of target gene expression in a target sample which is relative to a reference sample normalized to a reference gene.

### Western blot analysis

Total protein (30 μg) from myoblast cell lysate was separated via SDS-PAGE electrophoresis and then transferred to Immobilon^®^ PVDF membranes using the protocols outlined in our previous reports [32, 65, 66]. The membranes were blocked in TBST (TBS with 0.1% Tween 20) with 5% skim milk at room temperature for 1 h and then incubated overnight in primary antibodies at 4°C. The membranes were then incubated in TBST with secondary antibodies at room temperature for 1 h. The proteins were imaged on an Immobilon chemiluminescent HRP substrate using an ImageQuantTM LAS 4000 biomolecular imager (G.E. Healthcare, Little Chalfont, UK).

### Immunofluorescence staining

Immunofluorescence staining was performed following the protocol outlined in our previous reports [32]. Briefly, the cells were fixed in 3.7% paraformaldehyde at room temperature for 30 min, permeabilized with 0.1% Triton™ X-100 for 10 min and blocked with 1% bovine serum albumin (BSA) at room temperature for 1 h, prior to labeling with rabbit anti-desmin primary antibodies (1:500) and mouse-anti Pax-7 primary antibodies (1:100) at 4°C overnight. The cells were then washed twice using 1X PBS and incubated with Alexa Fluor 488 goat anti-rabbit (1:1000) and Alexa Flour 594 rabbit anti-mouse (1:1000) secondary antibodies at room temperature for 1 h. Finally, the cells were then washed twice before counterstaining the nuclei with DAPI for 10 min. Images were captured using Nikon ECLIPSE TE300 Inverted Microscope (Nikon Tokyo, Japan). The total number of immunofluorescent positive staining cells in each group was counted and quantified by ImageJ software (*n* = 3 per group).

### Immunohistochemical (IHC) staining

Sections of skeletal muscle tissue were embedded in paraffin and then rehydrated and stained using a hematoxylin and eosin (H&E) kit and IHC Kit (Sigma-Aldrich, St. Louis, MO, USA) in accordance with the manufacturer’s instructions.

### Bioinformatics screening of direct miRNA targets of Pax-7 expression

Analysis of miRNAs binding to the 3′UTR of Pax-7 was performed using publicly available bioinformatics software (TargetScan: https://www.targetscan.org; miRWalk: mirwalk.umm.uni-heidelberg.de; miRDB: https://mirdb.org/). 12 miRNAs were identified (miR-133b-3p, miR-185-3p, miR-206-3p, miR-342-5p, miR-499-3p, miR-742-3p, miR-1194, miR-133c, miR-1949, miR-3078-3p, miR-3475-3p, and miR-5127) that could bind most effectively with Pax-7.

### Statistical analysis

Statistical analysis included unpaired Student’s *t*-tests in the comparison of two groups and one-way analysis of variance (ANOVA) tests involving comparisons of more than two groups. Results are presented as the mean ± standard deviation (SD) of at least three independent experiments. Between-group differences were considered significant if the *p*-values were less than 0.05.

## Supplementary Materials

Supplementary Table 1
