# Supplementary material for: High-mobility group box-1 impedes skeletal muscle regeneration via downregulation of Pax-7 synthesis by increasing miR-342-5p expression
Source: Aging (Albany NY). 2023 Nov 13;15(21):12618–32. doi: 10.18632/aging.205202 (PMC10683625; doi:10.18632/aging.205202)
Supplement: Supplementary Table 1 [file aging-15-205202-s001.pdf]

SUPPLEMENTARY TABLE

Supplementary Table 1. Primers sequences for qRT-PCR.

Genes	Forward (5′–3′)	Reverse (5′–3′)
Pax-7	GGTCCCCAGGATGATGAGA	TTGATGAAGACCCCACCAAG
GAPDH	ACCACAGTCCATGCCATCAC	TCCACCACCCTGTTGCTGTA
miR-185-3p	AGGGGCTGGCTTTCCTCTGGT	TGGTGTCGTGGAGTCG
miR-206-3p	TGGAATGTAAGGAAGTGTGTGG	TGGTGTCGTGGAGTCG
miR-342-5p	AGGGGTGCTATCTGTGATTGAG	TGGTGTCGTGGAGTCG
miR-499-3p	GAACATCACAGCAAGTCTGTGCT	TGGTGTCGTGGAGTCG
miR-742-3p	GAAAGCCACCATGCTGGGTAAA	TGGTGTCGTGGAGTCG
miR-1194	TCCTAGATCGTCAATGAGTAAG	TGGTGTCGTGGAGTCG
miR-133c	TTTGGTCCCCTTCAAGGAGTCAG	TGGTGTCGTGGAGTCG
miR-133b-3p	TTTGGTCCCCTTCAACCAGCTA	TGGTGTCGTGGAGTCG
miR-1949	CTATACCAGGATGTCAGCATAGTT	TGGTGTCGTGGAGTCG
miR-3078-3p	TTGCTGGGGTAGTCTTTAGG	TGGTGTCGTGGAGTCG
miR-3475-3p	TCTGGAGGCACATGGTTTGAA	TGGTGTCGTGGAGTCG
miR-5127	TCTCCCAACCCTTTTCCCA	TGGTGTCGTGGAGTCG
U6 snRNA	CTCGCTTCGGCAGCACA	AACGCTTCACGAATTTGCGT
